# A Short Review on the Valorization of Green Seaweeds and Ulvan: FEEDSTOCK for Chemicals and Biomaterials

**DOI:** 10.3390/biom10070991

**Published:** 2020-07-02

**Authors:** D. Shanthana Lakshmi, Sivashunmugam Sankaranarayanan, Tejal K Gajaria, Guoqiang Li, Wojciech Kujawski, Joanna Kujawa, Rodrigo Navia

**Affiliations:** 1 RO Membrane Division, CSIR-Central Salt and Marine Chemicals Research Institute (CSMCRI), G.B. Marg, Bhavnagar 364002, Gujarat, India; 2 Scientific and Technological Bioresource Nucleus (BIOREN), Universidad de La Frontera, Av. Francisco Salazar 01145, Temuco 4780000, Chile; sivashunmugams@gmail.com (S.S.); rnaviadiez@gmail.com (R.N.); 3 Division of Applied Phycology and Biotechnology, CSIR-Central Salt and Marine Chemicals Research Institute (CSMCRI), G.B. Marg, Bhavnagar 364002, Gujarat, India; Tejalgajaria@gmail.com; 4 Academy of Scientific and Innovative Research (AcSIR), Ghaziabad 201002, Uttar Pradesh, India; 5 Nicolaus Copernicus University in Toruń, Faculty of Chemistry, 7, Gagarina Street, 87-100 Toruń, Poland; grantli@doktorant.umk.pl (G.L.); wkujawski@umk.pl (W.K.); 6 Department of Chemical Engineering, Faculty of Engineering and Sciences, Universidad de La Frontera, Av. Francisco Salazar 01145, Temuco 4780000, Chile; 7 Centre for Biotechnology and Bioengineering (CeBiB), Universidad de La Frontera, Av. Francisco Salazar 01145, Temuco 4780000, Chile

**Keywords:** green seaweed, ulvan, sulfated polysaccharides, chemicals, value-added biomaterials

## Abstract

This short review analyzed the recent trend towards, progresses towards the preparation of chemicals of, and value-added biomaterials from marine macroalgae resources, especially green seaweeds and their derived ulvan polysaccharides for various applications. In recent years, ulvan both in pristine and modified forms has gained a large amount of attention for its effective utilization in various areas due to its unique physiochemical properties, lack of exploration, and higher green seaweed production. The pristine form of ulvan (sulfated polysaccharides) is used as a bio-component; food ingredient; or a raw material for the production of numerous chemicals such as fuels, cosmetics, and pharmaceuticals, whereas its modified form is used in the sector of composites, membranes, and scaffolds, among others, because of its physicochemical properties. This review highlights the utilization of green seaweed and its derived ulvan polysaccharides for the preparation of numerous chemicals (e.g., solvents, fuel, and gas) and also value-added biomaterials with various morphologies (e.g., gels, fibers, films, scaffolds, nanomaterials, and composites).

## 1. Introduction

Green tides are large accumulations of green seaweeds that are associated with the eutrophication of the marine environment, mainly referring to coastal waters and estuaries. The macroalgal blooms import detrimental effects on the water chemistry, ecosystem, environment, and economy of coastal areas [1,2]. Green tides have been receiving increasing attention around the world. Thus, many investigations on their cause, consequence, and applications have been conducted [3,4,5,6]. It is important to tackle the problems resulting from green tides by mitigating the growth of green seaweeds and utilizing algae-based products. Currently, the development and utilization of sustainable biofuels have obtained abundant concern in the world due to the growing population and energy demand, depleting fossil fuel reserves, global warming, and deteriorating environment [7]. Algae are third-generation biomass and their potential as a feedstock for biofuel production is intensely growing. The utilization of macroalgae [8,9] and microalgae [10] as feedstock for the production of biofuels such as bioethanol, biodiesel, and biohydrogen offers several advantages, compared with the utilization of first- and second-generation biomass feedstocks [11]. For instance, seaweeds possess a high level of structural polysaccharides and low lignin contents. In addition to serving as feedstocks for biofuel production, green seaweeds are also an important source of high-value chemicals, such as polyunsaturated fatty acids, carotenoids, phycobilins, and polysaccharides [12]. Most importantly, green seaweeds are a cheap and important source for biomaterials. For instance, ulvan is a sulfated polysaccharide that can be extracted from green seaweeds. Ulvan has been considered as an attractive material for food, pharmaceutical, agricultural, and medical applications due to its varying physicochemical properties and important biological activities [13].

**Figure 1:**
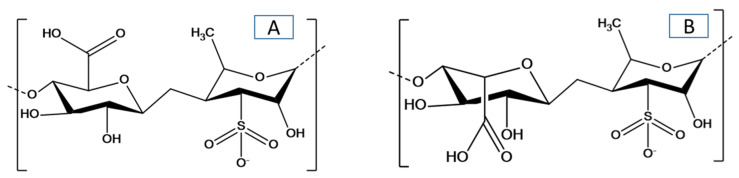
Chemical structure of ulvan with the major repeating disaccharide units: (**A**) glucuronic acid and rhamnose 3-sulfate, and (**B**) iduronic acid with rhamnose 3-sulfate.

Some researchers have reviewed the chemistry and biological activities of green seaweeds [4]; the extraction, structure, composition and function properties of ulvan [14,15]; and the applications of ulvan as a constituent of hybrid biomaterials [16]. Ulvan mainly consists of sulfated rhamnose, glucuronic acid, iduronic acid, and xylose [14]; the structure of ulvan with the major repeating disaccharide units is shown in Figure 1. The main objective is to highlight recent advances in the field of green seaweed and ulvan polysaccharides. This review discusses the direct utilization of green seaweeds in the production of biofuels and other high-value materials or chemicals (e.g., proteins, lipids, fatty acids, oils, proteins, natural pigments, antioxidants, and biological components) along with the development of ulvan-based materials and their applications. The future direction of the utilization of green seaweeds and the preparation and application of ulvan-based materials will be addressed. To the best of our knowledge, it is the first time there has been a review and discussion of the valorization of green seaweeds and their derived ulvan polysaccharides.

## 2. Green Seaweeds and Their Applications

Species of green seaweed from the Genus *Ulva* (Phylum Chlorophyta, Class Ulvophyceae, Order Ulvales, Family Ulvaceae) have high growth rates and productivities. This type of seaweeds is easily accumulated in coastal areas around the world. The green seaweeds consist of ≈36% carbohydrate, ≈11% protein, and ≈53% ashes and rich in minerals [4,6]. Green seaweeds (*Ulva* species) have some unique qualities, such as high growth rates irrespective of geographical location/season and high polysaccharide content [5]. Most importantly, compared to terrestrial sources, the growth of green seaweeds is faster and it does not require agricultural land/inputs (fertilizer, pesticides, and water), having a possibility for large-scale potential production [11,17]. The growth of green seaweeds does not compete with agricultural crops for land and water, which can tackle the conflict between food and fuels compared with corn and sugarcane feedstocks [11,17]. Moreover, green seaweeds possess a rapid reproduction rate and high growth rate, which makes them able to be harvested more than once in a year [9]. Therefore, green seaweeds are potential feedstocks for the production of biofuels, biomaterials, and high-value chemicals.

### 2.1. Biofuel Production from Green Seaweeds

The utilization of green seaweeds as feedstocks for the production of biobutanol has gained significant interest in science and been reported in the literature [18]. In this section, the utilization of green seaweeds in the acetone, butanol, and ethanol (ABE) process will be briefly discussed. Van der Wal et al. [19] used *Ulva lactuca* for ABE fermentation. Firstly, a hydrolysate containing 75–93% of the sugars from *Ulva lactuca* was prepared by using pretreatments and enzymatic hydrolysis of *Ulva* without chemical catalysts. Subsequently, the hydrolysate was used for the production of ABE by using *Clostridium acetobutylicum* and *Clostridium beijerinckii*. It was found that a high yield of 0.35 g ABE/g sugar was achieved in this process. More interestingly, *Clostridium beijerinckii* produced 1,2-propanediol from rhamnose. *Clostridium acetobutylicum* produced mostly organic acids. The obtained results demonstrate a great potential of *Ulva lactuca* as a feedstock for ABE fermentation [19]. Potts et al. [20] also reported butanol production from *Ulva lactuca* by using ABE fermentation. The *Ulva lactuca* seaweeds were firstly manually and mechanically harvested, dried, and ground. Subsequently, the acid hydrolysis of ground algae was conducted to extract carbohydrates for the algal sugar solution preparation. Finally, butanol was produced by the fermentation of the prepared algal sugar solution using *Clostridium beijerinckii* and *Clostridium saccharoperbutylacetonicum*. It was found that the butanol concentration in the fermentation broth was 4 g/L, and the removal of the excess solids from the hydrolysate before fermentation resulted in a 75% increase in productivity [20]. Both examples demonstrate the possibility and potential of the utilization of green seaweeds as feedstocks for the production of biobutanol through ABE fermentation.

Margareta et al. [21] reported biohydrogen production by using dark fermentation from algal biomass *Ulva* sp. for the first time. The acid–thermal combined pretreatment was used to release fermentable sugars from the green algal biomass. The *Clostridium butyricum* CGS5 achieved the highest cumulative hydrogen production, equal to 2340 mL/L; the maximum hydrogen productivity, equal to (208.3 mL/L per hour); and hydrogen yield equal to 1.53 mol H_2_ per mol of reducing sugar. The hydrogen productivity in this study was better than the hydrogen productivity in Jung et al.’s work [22], in which *Laminaria japonica* was used as feedstock [21].

Besides the production of biobutanol and biohydrogen from green seaweeds, the production of biogas from green seaweeds is also reported in literature. Akila et al. [23] investigated the production of biogas and biofertilizer from green seaweed *Ulva* sp. by using the anaerobic digestion method. The *Ulva* sp. was mixed with organic matter (cow dung) for the biogas production. The solid residue was used as an organic fertilizer for the growth of mung bean. Their research provided an example of maximizing the utilization of green seaweeds in an ecofriendly and sustainable way [23]. Mhatre et al. [24] explored strategies for improving biogas production from green seaweed *Ulva lactuca*. The individual and sequential extractions were conducted to investigate the influence of removal of sap, which contains valuable minerals that are essential for growth of agricultural crops, ulvan, and protein on methane yields. The biomethane production was enhanced after the extraction treatments, and the highest methane yield (408 mL/g) was obtained in the sap and ulvan-removed residue. This was because high protein and sulfate content are major inhibitors in anaerobic digestion of *Ulva lactuca*. The extractions prior to anaerobic digestion not only improved the methane productivity, but also provided high-value products such as sap, protein, and ulvan. The proposed strategy makes the biomethane production process more efficient and sustainable [24].

Biodiesel can be an alternative and efficient fuel to replace the use of fossil fuels. It is derived as monoalkyl esters of long-chain fatty acids and is usually produced from algal oils, waste cooking oil, and edible and non-edible oils [25]. Kalavathy et al. [26] used *Ulva lactuca* as feedstock for the production of biodiesel and silica doped with zinc oxide as a heterogeneous nanocatalyst for the transesterification process. The lipid content of algal biomass was extracted by an autoclave followed by the ultra-sonication method, and the extraction of oil was carried out in the Soxhlet apparatus using solvent mixture of n-hexane and methyl tertbutyl ether. It was found that the maximum oil was extracted at optimal conditions of 5% moisture content of algal biomass, 0.15 mm size of biomass, and solvent/solid ratio of 6:1 at 55 °C in 140 min. The maximum biodiesel yield of 97.43% was obtained at optimized conditions of 800 °C calcination temperature, 8% catalyst concentration, 9:1 methanol to oil ratio, 55 °C reaction temperature, and 50 min reaction time [26]. Khan et al. [27] used *Ulva fasciata* as feedstock for the production of biodiesel. The oil was extracted with n-hexane and the transesterification was carried out by fast stirring using a 9:1 molar ratio of methanol/oil in the presence of waste industrial catalysts for 6 h at 80–100 °C. It was found that the maximum yield of biodiesel equal to 88% was achieved by using the waste brown dust from the steel converter as a catalyst in the transesterification process.

The aforementioned examples demonstrate the possibility and potential of the utilization of green seaweeds as feedstocks for the production of biobutanol, biohydrogen, biogas, and biodiesel. The seaweed hydrolysis methods, such as acid and enzymatic hydrolysis, are crucial to the preparation of sugar solution from seaweeds, which consequently affects the productivity of biobutanol and biohydrogen. The types of bacteria strains are also very important to the productivity of biobutanol and biohydrogen. In the biogas production process, the extraction treatments on seaweeds can enhance methane productivity due to the removal of inhibitors existing in the components of green seaweeds, such as ulvan. In the biodiesel production process, the oil extraction methods and the types of catalysts are crucial to biodiesel productivity because both factors can influence the efficiency of the transesterification process.

### 2.2. Green Seaweed-Derived Adsorbents

Nowadays, using algae to remove metals is a rapid, reversible, economical, and ecofriendly method [28,29,30]. Kumar et al. [28] used green algae *Ulva fasciata* sp. as adsorbent to remove copper from its aqueous solution. The copper adsorption behavior of *Ulva fasciata* sp. was investigated at different pH values, contact time, initial copper and adsorbent concentrations, and adsorbent size. It was found that the optimum pH value was 5 and the maximum adsorption capacity was 26.88 mg/g [28]. Ebrahimi et al. [31] modified the green algae *Cladophora sericioides* by L-cysteine to enhance the copper adsorption. The green seaweeds were modified by 7 days cultivation in a reactor containing 20 L of seawater and 3 mg/L-cysteine under illumination cycle 12/12 at an average intensity of 2500 lx at 25 °C. It was found that 65% and 95% of copper ions with initial concentration of 20 mg/L was removed by simple and modified algae, respectively. The maximum adsorption capacities were 13 and 19 mg/g for the simple and modified algae, respectively. The modification significantly improved the copper adsorption capacity [31].

El-Sikaily et al. [29] utilized dried green algae *Ulva lactuca* and its activated carbon to remove the toxic hexavalent chromium ions from aqueous solution, saline water, and wastewater. It was found that the maximum efficiencies of chromium removal were 92% and 98% for *Ulva lactuca* and its activated carbon, respectively. The maximum adsorption capacity was found to be 10.61 and 112.36 mg/g for dried green alga and the activated carbon developed from it, respectively. *Ulva lactuca* and its activated carbon are valuable materials for the removal of chromium from waters [29]. Al-Homaidan et al. [32] also used dry green algae as a adsorbent to remove hexavalent chromium Cr(VI) from aqueous solution. It was found that the pH significantly affects the adsorption capacity. *Cladophora glomerata* showed the maximum of 66.6% removal of Cr(VI) when 1.0 g dried algae was used in 100 mL aqueous solution containing an initial Cr(VI) concentration of 20 mg/L at 45 °C and pH 2 for 60 min of contact time. The maximum adsorption capacity was 1.32 mg/g. These results indicate that *Cladophora glomerata* is a potential material for Cr(VI) removal from aqueous solution [32].

Mwangi et al. [33] reported the performance of the seaweed (*Caulerpa serrulata*) before and after modification with ethylenediamine (EDA) on adsorption of copper, lead, and cadmium in aqueous solution. The adsorption capacities of EDA-modified seaweed for Cu, Cd, and Pb were 5.27, 2.12, and 2.16 mg/g, respectively, and the adsorption capacities of pristine seaweed for Cu, Cd, and Pb were 3.29, 4.57, and 1.06 mg/g, respectively.

Green seaweed-derived adsorbents can be used as low-cost adsorbents to remove metal ions such as copper, chromium, lead, and cadmium in aqueous solutions. Dried *Ulva lactuca* exhibits desirable adsorption capacities for copper ions and chromium ions. The active carbon derived from *Ulva lactuca* in particular has shown significantly increased chromium ion adsorption capacities and removal efficiency. Modification on the green seaweeds can improve the adsorption capacity of the green seaweed-based adsorbent.

### 2.3. Chemicals from Green Seaweeds

The seaweed biorefinery is a sustainable and economical way to fully utilize seaweeds for the production of high value products along with the production of biofuels. The various products obtained from seaweeds such as fatty acids, oils, proteins, natural pigments, antioxidants, and biological components are used in different areas such as food, cosmetics, therapeutics, and biofuels [34].

The extraction methods are critical for the production of high value products from seaweeds. Gullón et al. [35] summarized the extraction techniques of bioactive compounds such as polysaccharides, phenolic compounds, fatty acids, pigments, proteins, and vitamins from seaweeds and reviewed the application of algae and their extracts in meat products. Microwave-assisted extraction (MAE), ultrasound-assisted extraction (UAE), enzyme-assisted extraction (EAE), pressurized liquid extraction (PLE), and supercritical fluid extraction (SFE) have been used to improve the extraction efficiency and to preserve the quality of the final compounds [35].

Gajaria et al. [17] integrated the extraction of proteins with recovery of other high value compounds such as seaweed sap, total lipids, ulvan, and cellulose to utilize green seaweed *Ulva lactuca* effectively. The extraction processes were conducted in the following order: (1) sap extraction, (2) total lipid extraction, (3) ulvan extraction, (4) protein extraction, and (5) cellulose extraction. Different sap extraction procedures were applied to investigate their influence on the sap composition. It was found that the protein content extracted was 11% on dry weight basis with recovery efficiency of 68.75%. The in vitro digestibility of the protein extracts from green seaweeds was tested by using *o*-phthalaldehyde OPA assay [36] and the high digestibility equal to 85.86% indicated that the recovered protein from green seaweeds is suitable for food supplements. Biorefinery utilizes biomass components that are synthesized as a function of complex photosynthesis and therefore they have to be effectively processed to obtain the products along with bio-energy. The described biorefinery model improves the utilization efficiency of green seaweeds since the valuable products from each extraction process can be applied in different areas [17].

Trivedi et al. [37] proposed an integrated process consisting of four extraction process and one fermentation process to fully exploit biomass of the green seaweed *Ulva faciata*. Mineral rich liquid extract, lipid, ulvan, and cellulose were sequentially recovered by the extraction processes. In addition to the economically important chemical feedstocks, bioethanol was produced through the enzymatic hydrolysis and fermentation of the final cellulose fraction. The yield of ethanol is comparable to those obtained by direct processing of the individual components from primary biomass. It is believed that this integration of ethanol production and chemical feedstock recovery from green seaweeds could substantially enhance the sustainability of marine biomass utilization [37].

Besides the biorefinery approach, some value-added chemicals can be directly extracted from green seaweeds. Mzibra et al. [38] extracted polysaccharides from green seaweeds *Ulva rigida* and *Codium decorticatum* by using the hot water extraction method under neutral conditions. The extracts were used as biostimulants of tomato seed germination and plant growth. It was found that the compounds in the extract from green seaweeds increased seed germination percentage, plant biomass, and the content of chlorophylls *a* and *b*. The polysaccharide extract from green seaweeds can be used as a feasible and cost-effective biostimulants for plant growth [38].

Doh et al. [39] isolated cellulose nanocrystals (CNCs) from green seaweeds *Ulva lactuca* by applying a process consisting of depolymerization, bleaching, acid hydrolysis, and mechanical dispersion. It was found that the CNCs from seaweeds possess rod shape, good thermal stability, and high crystallinity. It is suggested that CNCs from seaweeds have the potential to be used to increase the mechanical properties of polymer materials for food packaging [39]. Sucaldito and Camacho [40] isolated CNCs from freshwater green seaweeds *Cladophora rupestris* by using hyrobromic acid hydrolysis. The physicochemical properties of CNCs were characterized. It was found that CNCs possess high crystallinity index equal to 94% and high thermal decomposition temperature equal to 381.6 °C. The isolated CNCs were incorporated into starch-based films prepared by solution casting and evaporation method. The mechanical strength of CNC-incorporated starch-based films was found to be significantly improved by 78% when the weight ratio of starch to CNCs was 100:1 [40].

In addition to the valuable products extracted from green seaweeds, some researchers used green seaweed extract in nanoparticle synthesis because the algal extract possesses natural reductants such as pigments and antioxidants [41,42,43]. For instance, Ishwarya et al. [44] synthesized zinc oxide nanoparticles by using the extract of green seaweed *Ulva lactuca*. The *Ulva lactuca* extract was used as a reducing and capping agent and mixed with zinc acetate solution for zinc oxide nanoparticle preparation. In comparison to the conventional synthesis methods of nanoparticles, the synthesis of nanoparticles by using green seaweeds as reducing agents is a green and environmentally friendly method [42].

As it is shown in Table 1, green seaweeds are versatile feedstocks for the production of biofuels and value-added products. It is of great importance of economy and environment to develop new and sustainable approaches such as biorefinery [45] to fully exploit the potential of green seaweeds.

## 3. Ulvan-Based Biomaterials and Their Applications

**Figure 2:**
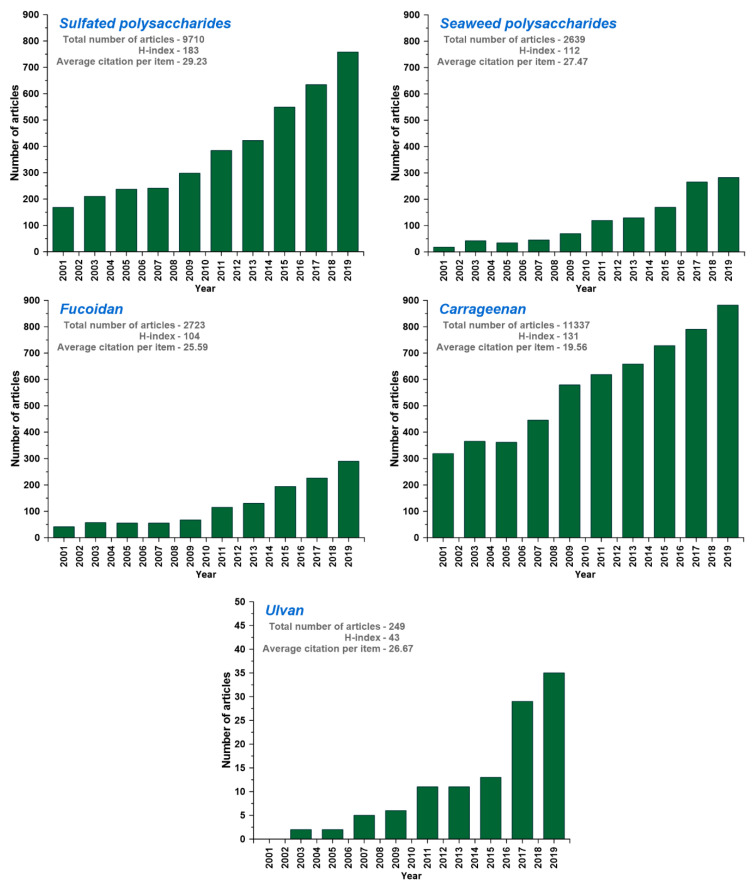
Institute for Scientific Information (ISI) Web of Science database for the articles published with the search topic of sulfated polysaccharides, seaweed polysaccharides, carrageenan, fucoidan, and ulvan within the 2001–2019 period (based on 3rd June, 2020 data).

In recent years there has been a sudden surge in interest to develop and utilize these types of under-exploited marine sources for novel materials. For instance, ulvan possesses attractive physicochemical properties and biological activities, resulting in its applications in different innovative applications [16,31]. According to the Web of Science database, distribution of research effect across green seaweed polysaccharides and number of research papers on the subject have increased. In the first 10 years of 21st century, the number of articles related to sulfated polysaccharides, seaweed polysaccharides, and ulvan barely changed in every year. From 2009 to 2019, the number of articles related to sulfated polysaccharides, seaweed polysaccharides, and ulvan increased from 300, 100, and 5 to 800, 300, and 36, respectively (Figure 2). However, the applications of ulvan retrieved less number of hits in the Institute for Scientific Information (ISI) Web of Index database in comparison with other materials, which shows the need and research opportunity to explore it for diverse applications. Although various seaweed-sulfated polysaccharides are known for their applications, limited literature are available for ulvan (Figure 2).

Ulvan is a polysaccharide extracted from cell walls of green seaweeds belonging to *Ulvales*, which generally accounts for 9–36% dry weight of the biomass *Ulva* [47,48]. Ulvan is mainly composed of sulfated rhamnose, glucuronic acid, iduronic acid, and xylose [14,47,49,50]. Rhamnose is of interest for its effect on biosynthetic pathways in the dermis and on plant immunity. Uronic acids (glucuronic and iduronic acids) and their sulfate esters are important constituents in mammalian glycosaminoglycans [14]. In addition, ulvan possesses a repeating disaccharide structure comprised of a uronic acid linked to a sulfated neutral sugar. Therefore, ulvan is a potential candidate for the applications in biomaterial science, for example in wound dressings and tissue engineering, as well as in pharmaceutical and biomedical applications due to its antioxidant activities, biological activities, and peculiar composition and structure [15,51].

The investigations on the structure, composition, physicochemical and functional properties, rheology, and gelling properties of ulvan are essential to explore the applications of ulvan [15,47,50,52,53,54]. The methods of extracting ulvan from green seaweeds have been reported in the literature [51,55,56,57]. Kidgell et al. [14] reviewed the methods of ulvan extraction from green seaweeds, the characterization of extracted ulvan, and their biological activities. During the extraction process, the subtle changes in pH lead to significant variation in ulvan yield as well as solubility of other macromolecules, which influences its biological functionalities [14]. Furthermore, the extract conditions affect the structure, thermal properties, and antioxidant properties of ulvan [58,59].

**Figure 3:**
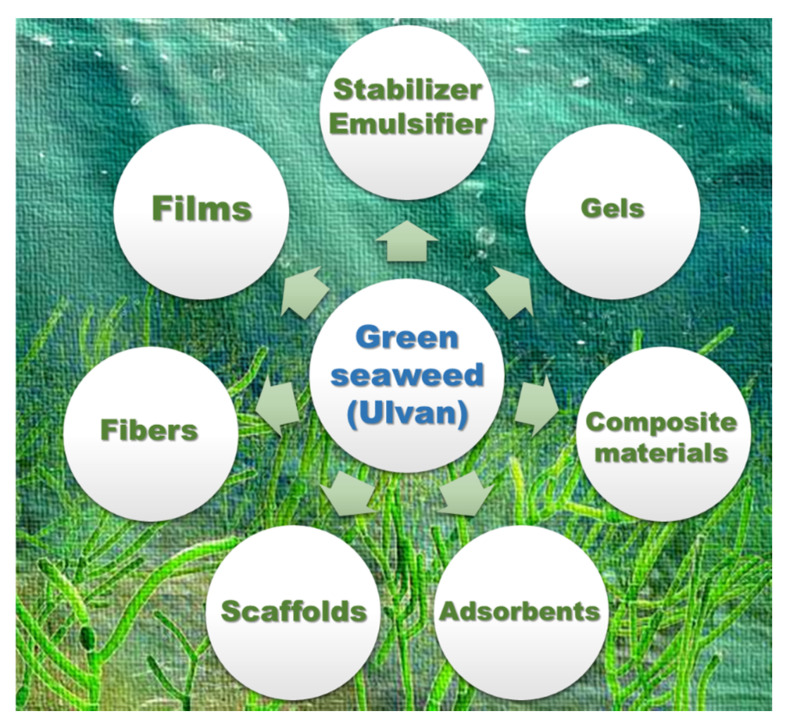
Possible bio-based materials using ulvan polysaccharides as a source.

Over the last decade, efforts have been attempted to improve life quality through biomaterials [60]. In general, ulvan polysaccharides are utilized for the preparation of certain biomaterials (Figure 3). In this section, ulvan-derived biomaterials along with their applications are summarized.

### 3.1. Ulvan-Based Hydrogel

The use of natural polymers such as polysaccharides in biomedical applications possesses enormous potential due to their advantages over synthetic polymers. Besides their biocompatibility and biodegragability, polysaccharides have a high number of functional groups that can be easily modified or tailored to provide desirable functional properties. Hydrogel made from polysaccharides has found its application in cell encapsulation, drug delivery, and scaffold for tissue engineering [61].

Haug [62] investigated the influence of borate and calcium on the gel formation of ulvan from *Ulva lactuca*. It was found that the ulvan hydrogel can be formed with the presence of borate and calcium ions in dialysis solution when the concentration of sulfated polysaccharide is no less than 1 wt % and the pH is above 7.5. The formation of borate–polysaccharide complexes create intermolecular linkages stabilized by calcium ions, which is critical to the gel formation from ulvan [62]. Lahaye and Axelos [50] investigated the gelling properties of water-soluble polysaccharides from green seaweeds (*Ulva* spp.). It was found that the investigated ulvan formed a weak hydrogel at a concentration of 1.6 wt % in deionized water. The elastic modulus of hydrogel increased significantly when boric acid and calcium chloride were added. In contrast to the observation of Haug [62], increasing pH to 7.5 and higher than 7.5 is detrimental to the gel formation, which might result from the different structure of ulvan from *Ulva* spp. [50]. Both studies found that the presence of borate and calcium ions is important to the formation of ulvan hydrogel.

Kanno et al. [63] reported the preparation and properties of the ulvan–chitosan polyion complex hydrogel. Ulvan was extracted from the green seaweed Chlorophyte *Ulva* sp. by using the hot water extraction method. The hydrogel was prepared from the mixture solution of ulvan and chitosan. The formed hydrogel possesses various characteristics such as high stability under acid and base conditions, weak influence on blood coagulation, and high adsorption of CuSO_4_, which makes it suitable as a biocompatible ion exchanger as well as other biocompatible materials. The prepared algal-based hydrogel is a renewable organic material and its preparation is simple and green [63].

Curcumin (Cur) is a hydrophobic polyphenolic compound that shows antioxidant, anti-inflammatory, anti-carcinogenic, and anti-cancer activities. However, its medical application is restricted because it is insoluble in water [64]. Bang et al. [64] modified the biocompatible ulvan with acetic anhydride to form amphiphilic polymers and prepared nanogels from acetylated ulvan by using dialysis method. The solubility of Cur in water was improved by 20,000 times by dispersing Cur into the prepared nanogels. It was concluded that the nanogel prepared from hydrophobically modified ulvan can be used to carry and deliver water-insoluble bioactive compounds [64].

Morelli et al. [65] grafted poly(N-isopropylacrylamide) chains onto the backbone of ulvan as a thermosensitive component. The thermosensitive hydrogel was prepared from the modified ulvan by dialysis method. The rheological properties and thermal behavior were investigated. It was found that the sol-gel transition of the prepared material occurred at 31 °C. The results showed that the prepared material is suitable for the in situ gelling systems in biomedical applications [65].

Morelli et al. [66]. prepared an in situ gelling material from ulvan by using enzymatically catalyzed crosslinking reactions. The ulvan was firstly modified with tyramine in order to make it recognizable by horseradish peroxidase enzyme (HRP). The combination of HRP with hydrogen peroxide catalyzed the gel formation through the covalent coupling the grafted phenols. Compared with chemical crosslinking, the enzymatic crosslinking is a straightforward, rapid, and clean method. The results obtained from biological investigations showed that the enzymatically crosslinked ulvan hydrogels are suitable used as vehicle for viable cells in the application of injectable cell delivery systems [66].

Morelli and Chiellini [61] prepared biodegradable hydrogel from functionalized ulvan by using photopolymerization under UV irradiation. Ulvan was functionalized by methacrylic anhydride or glycidyl methacrylate to add unsaturated groups in the ulvan structure. It was found that the UV-induced radical polymerization was not complete after 10 min due to the radical quenching activity of sulfated polysaccharides. This prepared material is suitable for cell encapsulation due to the antioxidant activity. Moreover, this partially crosslinked material is a good base for cytocompatible scaffolds because its softness promotes cell spreading [61].

Yoshimura et al. [67] prepared biodegradable superabsorbent hydrogels from ulvan by crosslinking ulvan with divinylsulfone (DVS) under alkaline aqueous condition. It was found that the maximal water absorbency was 80 g/g when 20 wt % relative to ulvan of DVS was added. The biodegradation speed of hydrogels was dependent on the amount of DVS [67].

The preparation of hydrogels from ulvan can be achieved by using the dialysis method; ulvan–chitosan polyion complex-gel formation; photopolymerization; and physical, chemical, and enzymatic crosslinking. The combination of ulvan modification and the gel formation method confers the ulvan-based gel desirable properties targeting various applications such as injectable cell delivery system, drug delivery, and scaffold for tissue engineering.

### 3.2. Membranes and Films

The use of biodegradable and active films for packaging has gained intensive attention due to environmental issues. The films based on polymers such as ulvan polysaccharides from natural renewable sources are non-toxic and environmentally friendly. Moreover, they possess antioxidant properties, which is vital for food packaging [58]. Therefore, Guidara et al. [58] developed active films based on ulvan that was extracted from the green seaweed *Ulva lactuca* by using acid extraction and enzymatic chemical extraction. The authors prepared 3 wt % ulvan solution by dissolving dried ulvan in distilled water followed by the addition of glycerol or sorbitol as plasticizer. It was found that enzymatic-chemical extraction showed more beneficial impacts on the optical, thermal, structural, and antioxidant properties of the prepared films. The films plasticized with glycerol exhibited better compact structure, lower temperature of transition, and greater antioxidant property than the films with sorbitol. The films prepared with ulvan seemed to be a promising packaging material due to their attractive optical, structural, thermal, and antioxidant properties [58]. Ganesan et al. [68] extracted ulvan from the green seaweed *Ulva fasciata* and utilized the extract to prepared edible films for food application. The films prepared from ulvan with glycerol significantly improved the physicochemical and mechanical properties of the films while decreasing the water vapor permeability, which is vital for food packaging. Most importantly, the ulvan polysaccharide-based films showed strong antioxidant activity, which is also very important to food packaging [68].

Wound dressings are widely used for the treatment of different wounds. Alves et al. [69] prepared polymeric membranes from crosslinked polysaccharide ulvan by solution casting for the application of medicated dressing. Firstly, ulvan was crosslinked by 1,4-butanediol diglycidyl ether (BDDE) to render the membrane insoluble in water and stable at physiological conditions. Subsequently, the pristine ulvan membranes and the crosslinked ulvan membranes incorporated with dexamethasone as model drug were prepared. It was found that the prepared membranes revealed remarkable water uptake ability and increased mechanical performance due to crosslinking. As the drug release results showed, 49% of the drug was released initially from membranes in 8 h. Afterwards, a slower release of the drug was detected and at day 14, wherein around 72% of dexamethasone was released from ulvan membranes. On the basis of these results, the prepared crosslinked ulvan membranes have great potential for use as drug delivery systems in medicated wound dressings [69].

Toskas et al. [70] synthesized ulvan, chitosan, and ulvan/chitosan polyelectrolyte membranes for the cultivation of osteoblasts. The combination of anionic ulvan and the cationic chitosan formed supramolecular structures and stabilized membranes due to the electrostatic interactions. The structure and porosity can be altered by changing the weight ratio of the two polysaccharides. The excellent attachment and proliferation of 7F2 osteoblasts on the prepared ulvan and ulvan/chitosan membranes were attributed to the nanofibrous structure mimicking the fibrous part of the extracellular matrix structure. It is concluded that the ulvan/chitosan membranes were potential materials for the development of scaffolds [70].

Bigot et al. [71] proposed a simple and an alternative procedure for the grafting of bioactive polysaccharide ulvan onto a polyvinyl chloride (PVC) surface. Firstly, isothiocyanate groups (NCS) were introduced onto the PVC surface using potassium isothiocyanate in a water/DMSO mixture. Subsequently, the polysaccharides were directly grafted onto the PVC-NCS surface using 1-ethyl-3-methyl-imidazolium phosphate ionic liquid as solvent for polysaccharides and as catalyst for the grafting reaction. The ulvan/PVC membranes have great potential in medical applications due to the wide application of PVC as biomaterial and the pharmacological activities of ulvan [71].

The ulvan film solubility in water and the water vapor permeability of ulvan film have been investigated by Gurdara et al. [72]. In their work, ulvan films were prepared from ulvan extracted from the green seaweed *Ulva lactuca* by varying the glycerol or sorbitol in specific plasticizer concentration. It was found that the increase of the plasticizers’ concentration in film resulted in a significant enhancement of the water solubility of films. The incorporation of glycerol into the composition of films showed a better performance on the enhancement of the solubility when compared to the films with sorbitol. On the contrary, the increase of the plasticizers’ concentration in film resulted in a decrease of water vapor permeability, and the incorporation of sorbitol into the composition of films showed a better performance on reduction of water vapor permeability compared with films with glycerol.

According to the aforementioned discussion, ulvan-based membranes and films have found their applications in food packaging, wound dressings, cultivation of osteoblasts, and in the medical field. The physicochemical properties of ulvan-based membranes and films can be tailored by plasticization with glycerol, crosslinking, and blending. In addition to the polymer solution casting method, the formation of ulvan membranes on a substrate made from synthetic polymers, for example, in PVC by surface grafting, could be a versatile method to combine the advantages of conventional synthetic polymers and natural polymers.

### 3.3. Nanofibers

Electrospinning is a versatile way to fabricate nanofibers from polymeric solution. The fabrication of fibers by using natural polymers for the applications in the biomedical area has received increasing attention due to their high porosity, high surface area-to-volume ratio, and architectural similarity to natural extracellular matrixes [73]. The poor rheological properties of the ulvan solution and the limited solubility of ulvan in various solvent systems restrict the formation of nanofibers from ulvan by using electrospinning. Thus, the improvement of the rheological properties and the charge-carrying capacity of the ulvan solution are important to fiber formation [16].

Toskas et al. [74] improved the spinnability of ulvan extracted from *Ulva rigida* by blending with poly(vinyl alcohol) (PVA) and successfully fabricated the ulvan-based nanofibers by electrospinning the ulvan/PVA solution. The prepared nanofibers had an average diameter down to 84 nm and a highly ordered crystalline structure. The spinnability of ulvan in combination with its biological and physicochemical properties can lead to new biomedical applications such as drug release systems [74]. Kikionis et al. [73] prepared novel composite nanofibers comprising ulvan and polycaprolactone (PCL) or ulvan and polyethylene oxide (PEO) by using an electrospinning technique. It was found that ulvan was incorporated well with the biocompatible polymers, providing the collective properties of these materials. The average diameters of the prepared fibers decreased when the ulvan content increased. The ulvan/PEO nanofibers can impart antithrombogenic properties and can be used as a drug release and wound healing medium. The ulvan/PCL nanofibers can be used as tissue engineering scaffolding materials due to the long biodegradation period of PCL [73].

### 3.4. 3D Porous Scaffolds

Scaffold design is crucial for tissue engineering applications. An ideal scaffold should possess suitable porosity and interconnectivity, mechanical properties, good biocompatibility, and biodegradability [75,76]. The sulfonated polysaccharide-based scaffolds for orthopedic tissue engineering were reviewed by Dinoro et al. [77].

The combination of both synthetic and natural materials can enhance the performance of these materials and broaden their applicability. Therefore, Alves et al. [75] combined ulvan with poly-D,L-lactic acid (PDLLA) to produce a novel scaffold for bone tissue engineering applications. The scaffolds of PDLLA loaded with ulvan particles were prepared by subcritical fluid sintering with carbon dioxide at 40 °C and 50 bar. Pristine ulvan particles and ulvan particles loaded with dexamethasone were dispersed within the PDLLA matrix. It was found that the prepared scaffold possesses appropriate morphologic features, suitable mechanical performance, and adequate cytocompatibility. Furthermore, the prepared scaffold can also be used as a drug delivery system [75].

Dash et al. [78] proposed an approach of producing photo-crosslinked polymeric ulvan scaffolds that are enzymatically treated for calcium phosphate deposition. Ulvan was firstly modified with methacrylic anhydride to obtain photoreactive groups, and subsequently the modified ulvan was used to prepare the photo-crosslinked scaffolds by using UV light. The crosslinked ulvan scaffolds were treated with alkaline phosphatase (ALP) to induce the mineral formation. It was found that the ulvan scaffolds were homogeneously mineralized at ambient temperature and the formed minerals contained apatite. The mineralized scaffolds were nontoxic and the formed minerals improved the osteogenic cell activity on the scaffolds. The biofunctionalized scaffolds could be potentially used as resorbable bone graft substitutes [78].

The polymer scaffolds possess high osteoconductivity and osteoinductivity when they are mineralized with apatite, which is very important to their medical applications. Therefore, Dash et al. [79] prepared ulvan-based scaffold from polyelectrolyte complexes of chitosan (40 wt %) and ulvan (60 wt %) and investigated the formation of apatitic minerals mediated by alkaline phosphatase (ALP). It was found that the calcium phosphate minerals were successfully deposited on the ALP-treated scaffold. The globular structure of the deposited minerals is beneficial to the cell attachment, proliferation, and extracellular matrix formation. In addition, the mineralized scaffolds are nontoxic. Therefore, it is a green way to fabricate scaffold from polyelectrolyte complexes. The prepared scaffold can be used as a resorbable materials for tissue engineering [79].

In addition to the ulvan-derived materials, ulvan was investigated as a stabilizing and emulsifying agent in colloidal formulation containing functional agents for food and cosmetic applications due to its edibility and amphiphilic character [80]. Ulvan was also used as a reducing and stabilizing agent for the synthesis of silver nanoparticles under mild conditions [81]. Furthermore, ulvan was used to prepare lysozyme/ulvan complexes to improve the antibacterial activity of lysozyme. The prepared lysozyme/ulvan can be used as a promising nanocarrier for positively charged bioactive molecules [13].

Additionally, their potential antioxidant activity has drawn tremendous attention to their applications in the cosmetic industry [14]. The sulfated polysaccharide containing extracts from *Ulva rigida* has been shown to protect HeLa cells from hydrogen peroxide-induced oxidative stress in vitro [82]. Furthermore, ulvans are rich in rhamnosyl residues, which are reported to promote cell proliferation and collagen biosynthesis, and the presence of glucuronic acids confers moisturizing properties that help to prevent skin damage from dry environments [83]. The unique chemical composition of ulvan provides distinct rheological properties and gelling characteristics to its suspensions at varying temperatures, pH values, and various cations, which enables ulvan to be used as a stabilizer and emulsifier in variety of applications including the food and cosmetics industries [15,80].

The peculiar structure, the strong bioactivity, and the presence of both hydrophilic (hydroxyl, carboxyl, sulfate) and hydrophobic (methyl) groups make ulvan a unique biopolymer for the preparation of functional materials. The reported literature on the preparation of ulvan-based materials, their characterization, and possible applications are summarized in Table 2.

## 4. Future Perspectives

Green seaweeds are very attractive feedstocks for biofuel production due to their high carbohydrate content. Dilute acid pretreatment is commonly used to obtain the fermentable sugar solution. However, the need for more environmentally friendly processes promotes the research on pretreatments using green solvents or enzymatic pretreatments. The development of the effective enzymes for seaweed hydrolysis and the bacteria strains for the fermentation process is highly needed to improve the efficiency of biofuel production. Prior to the fermentation process, the extraction of some components of seaweeds can enhance the biofuel production because these components of seaweed might inhibit the fermentation process [24]. However, these extracted components can be used as food ingredients, additives, cosmetics, fertilizers, and medicine. To fully utilize green seaweeds, the biorefinery process has been developed, which contains the extraction of high-value chemicals, such as proteins, lipids, fatty acids, oils, natural pigments, antioxidants, and biological components, and the fermentation process for biofuel production. The development of innovative, environmentally sustainable, and integrated processes is necessary to improve the economic feasibility of this biorefinery. In addition to the high-value chemicals and biofuel production from green seaweeds, the green seaweed-derived adsorbents can be used as environmentally friendly, sustainable, and low-cost adsorbents to remove heavy metal ions from water. The ability of seaweeds to accumulate nutrients [84], minerals [85], and metals [86] can be utilized for the bioremediation from polluted sites or of the wastewater sources. Studies are also accumulating for the use of removal of textile effluents as well as dying industries to minimize the environmental impact towards saving the aquatic life forms against the detrimental effects of pollution [87,88]. However, the modification methods and the method of preparing active carbon from green seaweeds need to be developed to improve the adsorption capability of the green seaweed-derived adsorbents.

To prepare novel biomaterials from green seaweed and its derived ulvan polysaccharides, the combination of various technologies is required. An integrated complex approach is required to achieve stable and efficient polysaccharide composites of different morphologies from ulvan polysaccharides, such as emulsions, thin films, nanoparticles, gels, nanofibers, and membranes [89]. Their bioactive properties are extending their exploration for utilization in various food packaging systems. Their biocompatibility and biodegradability guarantee their application in medical areas such as drug delivery and tissue engineering.

The application of green seaweed-based materials in membrane technology has not been fully explored. Membrane technology is a generic term used for different types of separation processes, which possess the capability to radically change the scenario of the water treatment and pollution control processes. The research on potential natural polymers that are able to substitute the conventional fossil-based polymers for membrane preparation is needed due to the growing environmental pollution. Polylactic acid, chitosan, and cellulose are commonly used biopolymers for membrane production [90]. However, very few studies are related to the membrane preparation from sulfonated polysaccharide. Sulfonated polysaccharide could be a potential polymer for the preparation of membranes due to their unique structure and composition. The hydroxyl and carboxyl groups make the sulfonated polysaccharide easily modified or crosslinked to improve their physicochemical properties to meet the requirements of the targeted membranes. The preparation of mixed-matrix membranes from ulvan, crosslinkers, and inorganic fillers also should be explored. In-depth investigation is required to explore ulvan as a potential membrane in various separation processes, such as micro- and ultrafiltration, pervaporation, and gas separation [91,92,93].

## 5. Concluding Remarks

Green seaweeds are emerging feedstocks for sulfated polysaccharide-based biopolymer products and are expected to gain many industrial interests in the near future. Even though there are challenges in the production of seaweed-based products, the preparation of biomaterials and chemicals from green seaweeds is still growing and the bioprocessing technologies for the utilization of green seaweeds are developing [94]. To make these technologies economically feasible, it is important to develop simple extraction routes with high yield and purity, improve the availability of seaweeds for operation, and develop downstream process to recover value-added materials. Considering the potential and unique chemical composition of green seaweeds, the exploitation of green seaweeds will make a great contribution to the development of sustainable and environmentally friendly chemical and fuel industries [95].

Due to the ever-growing demand upon pollution control and the minimization of petroleum-based products, bio-based materials have become a globally accepted option. Biomaterials occupy an indispensable position in material science, considering its availability, biodegradability, eco-friendly nature, and health-based applications. Biomaterials are commonly found in many applications from commodity to hi-tech due to the development of biotechnologies and the change of public awareness on environment. In recent years, various ulvan-derived materials have been developed, such as hydrogel, thin films, fibers, nanoparticles, and 3D porous scaffolds. Moreover, these ulvan-derived materials have found their applications in many areas, especially in medical applications, such as in drug delivery, wound dressings, and tissue engineering, due to the biodegradability and biocompatibility of ulvan. The exploration of ulvan-based materials as functional materials in other innovative applications such as in membrane technology is highly demanded.

The present review is intended to make comprehensive the possible applications practiced thus far with regards to green seaweeds and their chemical constituents including the sulfated polysaccharides, followed by a thorough discussion of the respective limitations. The wide array of applications of ulvan provides a deep insight into the chemical diversity of this natural polymer that allows us to devise possible tuning of its chemistry to analyze its suitability in other scientific domains such as regenerative medicines, whereas proteins with a proven mark of digestibility have presented enormous possibilities in the food and nutrition sectors. Thus, a well-designed cascading biorefinery out of the biomass generated by the cultivation practices operated on an industrial effluent or aquaculture farm would be a huge leap towards a techno-economically and environmentally sustainable seaweed farming, essentially contributing towards the development of the blue economy.

## Figures and Tables

**Table 1:** Green seaweed-derived products and their reported applications.

Type of Green Seaweed	Products	Preparation Methods	Applications	Reference
*Ulva lactuca*	Acetone, butanol, ethanol, 1,2-propanediol, and organic acid	Acetone-Butanol-Ethanol (ABE) fermentation by using *Clostridium acetobutylicum* and *Clostridium beijerinckii*	The possibility of using rhamnose-rich seaweeds as feedstock for 1,2-propanediol production	[19]
*Ulva lactuca*	Butanol	ABE fermentation by using *Clostridium beijerinckii* and *Clostridium saccharoperbutylacetonicum*	Biofuels	[20]
*Ulva* sp.	Bio-hydrogen	Dark fermentation by using *Clostridium butyricum CGS5*	Bioenergy	[21]
*Ulva lactuca*	Biodiesel	Transesterification process	Biofuel	[26]
*Ulva fasciata*	Biodiesel	Transesterification process	Biofuel	[27]
*Ulva* sp. mixed with cow dung	Biogas and bio-fertilizer	Anaerobic digestion	Organic fertilizer—for the growth of mung bean Biogas—as biofuel	[23]
*Ulva lactuca*	Biogas, sap, ulvan, and protein	Individual and sequential extractions Anaerobic digestion	High value chemicals Biofuels	[24]
*Ulva fasciata*	Dry solid material	The seaweed was used as adsorbent after washing, drying in sunlight, and cutting	The removal of copper from its aqueous solution	[28]
*Cladophora sericioides*	Modified composite form	The green seaweed was modified by L-cysteine and used as adsorbent	The removal of copper from its aqueous solution	[31]
*Ulva lactuca*	Activated carbon	The green seaweed activated carbon was prepared by using highly concentrated sulfuric acid	The removal of toxic hexavalent chromium ions from aqueous solution, saline water, and wastewater	[29]
*Ulva lactuca*	Biochar	The biochar was prepared by pyrolyzing the dried green seaweed at 300 °C for 2 h	Remediation of Remazol Brilliant Orange 3R in an up-flow fixed column	[46]
*Cladophora glomerata*, *Ulva intestinalis* and *Microspora amoena*	Dry solid material	The dry green seaweeds were used as adsorbent directly	The removal of hexavalent chromium Cr(VI) from aqueous solution	[32]
*Ulva lactuca*	Sap, lipids, ulvan, and protein	Seaweed biorefinery	The applications in food, cosmetics, therapeutics, and biofuels	[17]
*Ulva lactuca*	Cellulose nanocrystals (CNCs)	Depolymerization, bleaching, acid hydrolysis, and mechanical dispersion	The improvement of the mechanical properties of polymer materials for food packaging	[39]
*Cladophora rupestris*	Cellulose nanocrystals (CNCs)	Hyrobromic acid hydrolysis	The improvement of the mechanical strength of starch-based films	[40]

**Table 2:** Ulvan-derived biomaterials and their reported applications.

Source Materials	End Products	Preparation Method	Applications	Reference
Ulvan from *Ulva lactuca*	Hydrogel	The hydrogel was formed when dialyzed against seawater.	-	[62]
Ulvan from *Ulva* spp.	Hydrogel	The ulvan hydrogel was formed in distilled water and water containing borate and calcium ions.	-	[50]
Ulvan from *Ulva* spp.	Hydrogel	The hydrogel was prepared from the mixture solution of ulvan and chitosan.	Biocompatible ion exchanger as well as other biocompatible materials	[63]
Ulvan from *Ulva lactuca*	Hydrogel	Ulvan was modified with acetic anhydride to form amphiphilic polymers. Nanogels were prepared from acetylated ulvan by using the dialysis method.	Carrier and delivery of water-insoluble bioactive compounds	[64]
Ulvan from *Ulva* spp.	Hydrogel	The thermosensitive hydrogel was prepared from the modified ulvan with thermal-sensitive group by using the dialysis method.	In situ gelling systems in biomedical applications	[65]
Ulvan from *Ulva* spp.	Hydrogel	The thermosensitive hydrogel was prepared from modified ulvan by using enzymatically catalyzed crosslinking reactions.	Vehicle for viable cells in the application of injectable cell delivery systems	[66]
Ulvan from *Ulva armoricana*	Hydrogel	The biodegradable hydrogel was prepared from functionalized ulvan by using photopolymerization.	Cell encapsulation Cytocompatible scaffolds	[61]
Ulvan from *Ulva* spp.	Hydrogel	Hydrogels were prepared by crosslinking ulvan with divinylsulfone (DVS) under alkaline aqueous conditions.	-	[67]
Ulvan from *Ulva lactuca*	Film	Glycerol or sorbitol was used as a plasticizer. Film was prepared by casting solution into a plastic Petri disk.	Packaging material	[58]
Ulvan from *Ulva fasciata*	Film	Glycerol was used as a plasticizer. Film was prepared by casting solution in a framed glass plate.	Food packaging	[68]
Ulvan	Film	Film was prepared by casting solution in Petri dishes.	Drug delivery systems Medicated wound dressings	[69]
Ulvan/chitosan	Film	Film was prepared by casing solution on flat glass.	Cultivation of osteoblasts Potential materials for the development of scaffolds	[70]
Ulvan	Film	The ulvan film was formed by grafting of bioactive polysaccharide ulvan onto PVC surface.	Medical applications	[71]
Ulvan from *Ulva rigida*	Fiber	The ulvan-based nanofibers were prepared by electrospinning ulvan/PVA solution.	Drug release systems	[74]
Ulvan from *Ulva fasciata*/PEO	Fiber	The ulvan-based nanofibers were prepared by electrospinning ulvan/PEO solution.	Drug release and wound healing medium	[73]
Ulvan from *Ulva fasciata*/PCL	Fiber	The ulvan-based nanofibers were prepared by electrospinning ulvan/PCL solution.	Long-term drug release and tissue engineering scaffolding materials	[73]
Ulvan from *Ulva lactuca*/PDLLA	Scaffolds	The scaffolds of PDLLA loaded with ulvan particles were prepared by subcritical fluid sintering with carbon dioxide at 40 °C and 50 bar.	Bone tissue engineering applications	[75]
Ulvan from *Ulva armoricana*	Scaffolds	The ulvan scaffold was prepared by using photo-crosslinking.	Resorbable bone graft substitutes	[78]
Ulvan from *Ulva armoricana*	Scaffolds	The ulvan scaffold was prepared by the formation of ulvan–chitosan polyelectrolyte complexes.	Tissue engineering	[79]

Footnotes: PVC—polyvinyl chloride, PEO—polyethylene oxide, PVA—poly(vinyl alcohol), PCL—polycaprolactone, PDLLA—poly-D,L-lactic acid.
